# An Artificial Intelligence Prediction Model of Insulin Sensitivity, Insulin Resistance, and Diabetes Using Genes Obtained through Differential Expression

**DOI:** 10.3390/genes14122119

**Published:** 2023-11-23

**Authors:** Jesús María González-Martín, Laura B. Torres-Mata, Sara Cazorla-Rivero, Cristina Fernández-Santana, Estrella Gómez-Bentolila, Bernardino Clavo, Francisco Rodríguez-Esparragón

**Affiliations:** 1 Research Unit, Hospital Universitario de Gran Canaria Doctor Negrín, 35019 Las Palmas, Spain; lbtm1002@gmail.com (L.B.T.-M.); scazorla@ull.edu.es (S.C.-R.); cristina.fersan76@gmail.com (C.F.-S.); estrellagbentolila@gmail.com (E.G.-B.); bernardinoclavo@gmail.com (B.C.); 2 CIBER de Enfermedades Respiratorias, Instituto de Salud Carlos III, 28029 Madrid, Spain; 3 Department of Internal Medicine, Universidad de La laguna, 38296 La Laguna, Spain

**Keywords:** microarray, expression estimation, pathway analysis, machine learning

## Abstract

Insulin is a powerful pleiotropic hormone that affects processes such as cell growth, energy expenditure, and carbohydrate, lipid, and protein metabolism. The molecular mechanisms by which insulin regulates muscle metabolism and the underlying defects that cause insulin resistance have not been fully elucidated. This study aimed to perform a microarray data analysis to find differentially expressed genes. The analysis has been based on the data of a study deposited in Gene Expression Omnibus (GEO) with the identifier “GSE22309”. The selected data contain samples from three types of patients after taking insulin treatment: patients with diabetes (DB), patients with insulin sensitivity (IS), and patients with insulin resistance (IR). Through an analysis of omics data, 20 genes were found to be differentially expressed (DEG) between the three possible comparisons obtained (DB vs. IS, DB vs. IR, and IS vs. IR); these data sets have been used to develop predictive models through machine learning (ML) techniques to classify patients with respect to the three categories mentioned previously. All the ML techniques present an accuracy superior to 80%, reaching almost 90% when unifying IR and DB categories.

## 1. Introduction

Diabetes (DB) is a chronic disease that is characterized by presenting a set of metabolic disorders related to the appearance of chronic hyperglycemia, as well as alterations in the metabolism of carbohydrates, fats, and proteins, due to the existence of problems in the secretion and/or action of insulin [1]. This is because the pancreas does not produce insulin in a sufficient quantity or because the body itself is not capable of adequately using the insulin it generates, and consequently, the glucose is not assimilated by the cells and remains in the blood, where an increase in its concentration occurs [2]. DM has a high prevalence and incidence in the population and can cause significant health problems, leading to serious complications such as cardiovascular disease, stroke, blindness, and amputation of lower limbs, among others. In the case of gestational diabetes, it can cause problems during pregnancy, both for the mother and the fetus or newborn.

Some of the complications caused by diabetes can be avoided or delayed by developing preventive actions and good control. Although some risk factors cannot be modified, it is possible to develop preventive actions that avoid or delay their impact on the development of the pathology.

Currently, the prevalence of diabetes is increasing worldwide. About 463 million adults between the ages of 20 and 79 have diabetes. This represents 9.3% of the world population in this age group. The total number is projected to rise to 578 million (10.2%) by 2030 and 700 million (10.9%) by 2045. By 2030 and 2045, spending is forecast to reach USD 825 billion and USD 845 billion, respectively [3].

Type 2 diabetes (DM2), which accounts for 90–95% of all diabetes, is essentially characterized by pancreatic β-cell dysfunction in the presence of insulin resistance. Therefore, a compensatory increase in insulin production is required [4]. Insulin is a potent pleiotropic hormone that affects processes such as cell growth, differentiation, apoptosis, ion flow, energy expenditure, and carbohydrate, lipid, and protein metabolism [5]. These diverse actions are initiated by specific binding to high-affinity receptors on the plasma membrane of target cells [6,7], which then activate both a metabolic signaling pathway through PI-3 kinase and a mitogenic pathway through Ras/MAPK cascade. Insulin-mediated signaling has been studied extensively with respect to early events in gene translation. However, an understanding of the more distal events in insulin signaling involving multiple effector systems and the integrated effects on gene expression that underlie the hormone’s multiple actions is lacking. Nowadays, it is possible to conduct a comprehensive assessment of differential expression in response to insulin using microarray technology. This knowledge could improve the understanding of insulin action and how responses are integrated to mediate the spectrum of hormonal effects [5]. Skeletal muscle is the primary site for insulin-dependent glucose disposal in humans [8,9]. Insulin stimulates the uptake and use of glucose in oxidative and storage pathways. Approximately 80% of insulin-responsive glucose uptake affects skeletal muscles, and this tissue is the primary site of glycogen storage, lipid oxidation, protein turnover, and thermogenesis. Insulin resistance involving skeletal muscles is critical in the pathogenesis of human diseases, including metabolic syndrome and type 2 diabetes, causing a large and increasing public health burden [5]. We intend to analyze the genes related to the DB, IR, and IS possibilities regarding the insulin–diabetes binomial.

Although there are currently more sophisticated techniques, such as next-generation sequencing (NGS), microarray data analysis has been one of the most important successes in the interaction between statistics and bioinformatics in the last two decades [10,11].

On the other hand, in recent years, prediction techniques associated with machine learning (ML), such as K-nearest neighbors, neural networks, support vector machines, random forests, and cutting-edge techniques, such as deep learning (multilayer perceptron), have been developed in order to obtain predictive models with high accuracy.

The main objective was to obtain a predictive model using ML techniques that allow patients to be classified as IS, IR, or DB through differentially expressed gene expression data to predict future patients who are IS, IR, or who end up being diabetic.

## 2. Materials and Methods

**Figure 1:**
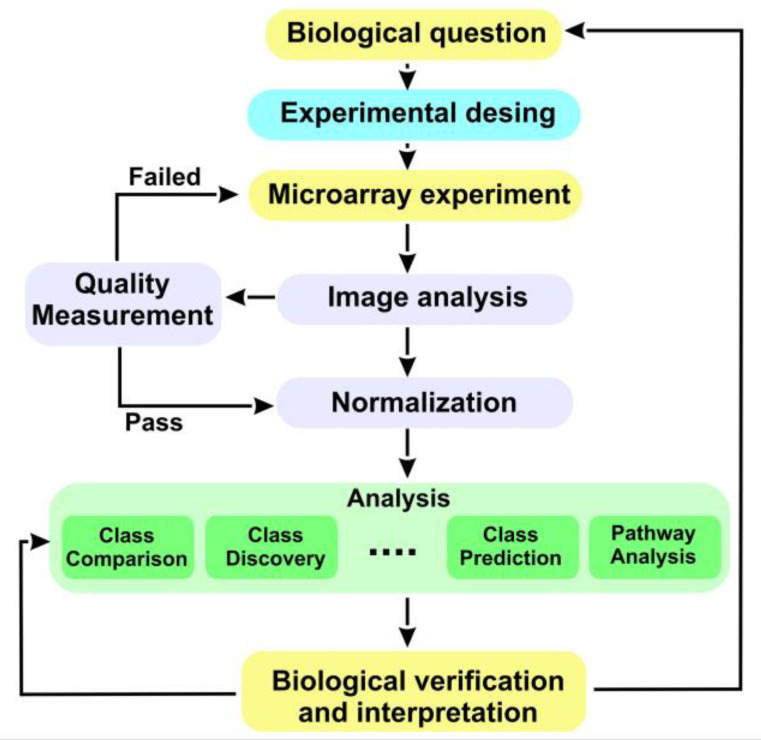
Microarray data processing [11].

**Figure A1:**
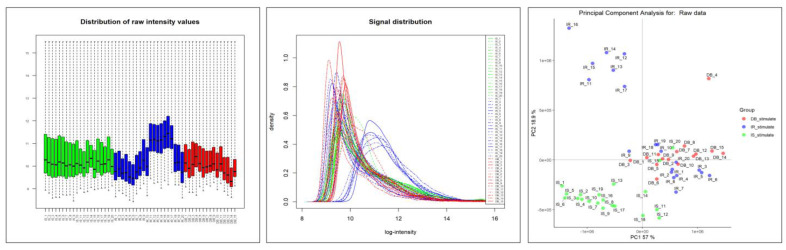
Result of the raw data analysis process.

**Figure A2:**
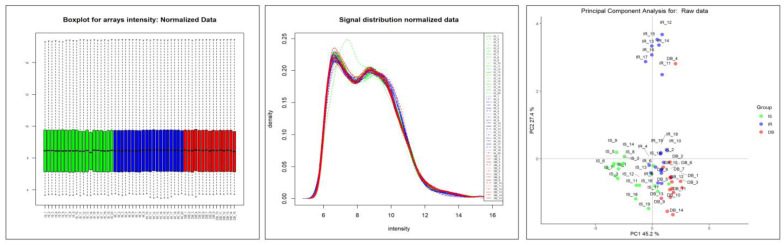
Result after normalizing the data.

**Figure A3:**
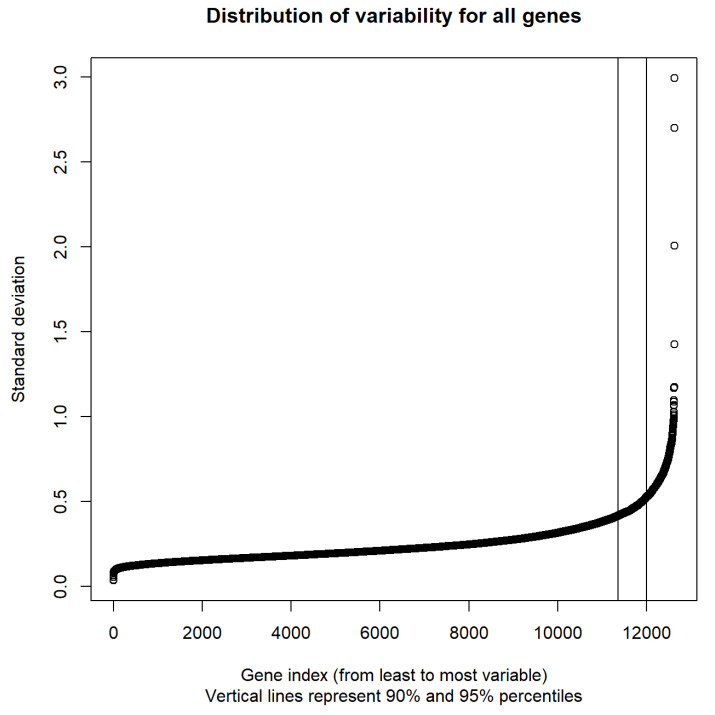
Distribution of variability of all genes.

**Figure A4:**
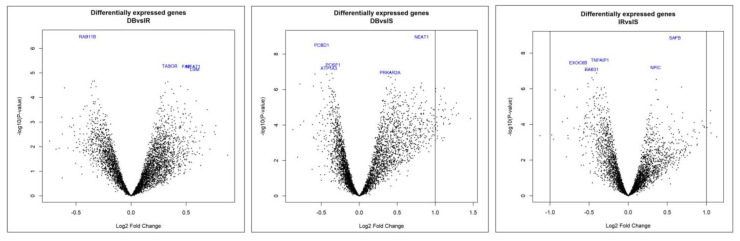
Volcano plot of the different comparisons.

**Figure A5:**
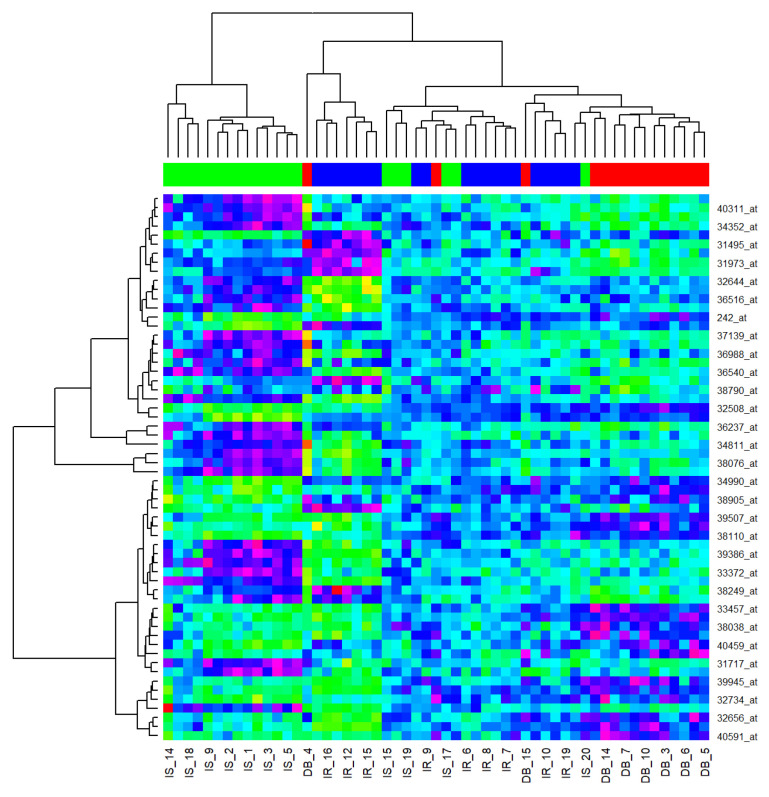
Heatmaps of differentially expressed genes grouped by similarity.

This work can be broken down into two parts. Microarray data analysis has been one of the most important hits in the interaction between statistics and bioinformatics in the last two decades. The analysis of microarray data can be performed in different ways using different tools [10]. In the first part, the classic microarray data analysis is carried out using the Bioconductor platform and the R program. The analysis has been based on data from a study deposited in Gene Expression Omnibus (GEO) with the identifier “GSE22309”. These data have been deposited in GEO following the MIAME20 (minimum information about a microarray experiment) standards. In this study, the information of the 55 patients was analyzed after having performed the technique of applying euglycemic hyperinsulinemic clamps. The information based on the microarray image was recorded in 55 files (20 files associated with IS, 20 with IR, and 15 with diabetics) type “.cel” (Cell Intensity File); cel files are the files with the “raw data” originated after microarray scanning and preprocessing using Affymetrix Human Genome U95A Array software [10]. Annotations for the Affymetrix Hu95A array model are found in the Bioconductor package hgu95av2.db [12]. With this data set, a classic microarray analysis has been performed through the R [13] Bioconductor [14] platform (https://www.bioconductor.org/) (accessed on 1 November 2021). After reading the file with the characteristics of the samples (targets), the steps in the microarray analysis process have been the following: (a) Quality control of the raw data. This step is very important since bad quality data could introduce much noise in the analysis that the normalization process could not solve; to check it, a multiple boxplot to visualize the intensity distribution of the arrays, a histogram of the signal density distribution, and principal component analysis were performed (Figure A1); (b) Normalization. Before beginning with differential expression analysis, it is necessary to make the arrays comparable among them and try to reduce and, if possible, eliminate all the variability in the samples not owing to biological reasons. The normalization process attempts to ensure that the intensity differences present in the arrays are due to the differential expression of genes rather than artificial biases due to technical problems. The robust multichip analysis (RMA) [15] method was used. This process consists of three discrete steps: background correction, normalization, and summarization); (c) Quality control of normalized data. After performing normalization, it is interesting to perform a quality control again to check how the data look. The same graphs as before have been performed with normalized data (Figure A2); (d) Identification of differentially expressed genes. If a gene is differentially expressed, there is expected to be a certain difference between the groups; therefore, the overall variance of the gene will be greater than that of those that do not have differential expression. Plotting the overall variability of all genes is useful to decide which percentage of genes shows variability that can be attributed to other causes than random variation [10] (Figure A3); (e) Filtering. Filtering out those genes whose variability can be attributed to random variation, that is, the genes that are reasonably not expected to be differentially expressed, has proven useful in reducing the number of tests to be performed with the corresponding increase in power [16]. A standard filter was applied that retains 50% of the genes with the greatest variability among those that were correctly annotated; (f) Selection of differentially expressed genes. This consists of performing some type of test, usually on a gene-wise basis, to compare gene expression between groups. This can be performed using many different approaches [17]. In this case, the linear models for the microarrays method, implemented in the “limma” package [18], were used to select differentially expressed genes. The comparisons between groups were DB vs. IR, DB vs. IS, and IR vs. IS. The adjusted *p*-value was calculated following Benjamini and Hochberg [19]; (g) Volcano plots of the genes most relevant to each were performed. A visualization of the overall differential expression can be obtained using volcano plots. These plots show if there are many or few genes with a large fold change and significantly expressed or if this number is low [10] (Figure A4); (h) The genes selected as differentially expressed were grouped to look for common expression patterns between experimental conditions using “heatmaps” (Figure A5); (i) Lists of differentially expressed genes were annotated in various databases (Entrez, Unigene, Gene Ontology, KEGG, etc.) using the affymetrix microarray annotation packages available in the Bioconductor project [14]. To contribute to the biological interpretation of the results, two types of enrichment analysis [20,21] or “gene set analysis” were carried out, which seek to establish whether the functional categories of the selected genes appear among these genes with more or less frequency than among all of the genes in the genome group. If so, it indicates that the list of genes is “enriched” in these functionalities, or what is the same as these are the processes affected by the differences; (j) The basic enrichment analysis is used as described in the works of Falcon and Gentleman [20] implemented in the Bioconductor GOstats [20] package. Analyses of this type require a minimum number of genes to be reliable, so all genes with adjusted *p*-values less than 0.05 were included (without filtering by minimum “fold-change”). Additionally, the basic enrichment analysis implemented in the ReactomePA [21] package from Bioconductor was also performed. In this case, given the small number of differentially expressed genes between the DB and IR categories, all genes were entered into the analysis for these two categories. In the other two comparisons, DB vs. IS and IR vs. IS, those genes that had an adjusted *p*-value less than 0.05 were included. A summary of the array process followed is shown in Figure 1.

One of the main objectives of this research was to translate the findings on differentially expressed genes into clinical applications. Thus, a machine learning approach was developed to check if these genes are suitable for predicting diabetic targets. Due to this, a second part was conducted, evaluating the prediction ability for selected genes using ML techniques. The classic procedure was followed. At first, a principal component analysis (PCA) was carried out with the 60 selected genes to see how they are grouped. The seven variables with standard deviation/eigenvalues greater than or very close to 1 were selected (max standard deviation 5.15 and min 0.995). These seven variables explained 83.4% of the total amount of the variance (the first, 44.18%, and the second, 25.66%). These variables were later used as an input data set to predict the target variable through a neural network.

For ML techniques, input data were randomly split into training data (train), made up of 70% of the 55 records (38 rows), and the remaining 30% (17 rows) were used as test data [22]. With the intention of reducing noise, each of the 60 selected genes was normalized using the following formula:

(1) $$Z=\frac{X-\min\left(X\right)}{\max\left(X\right)-\min\left(X\right)}$$

where *X* is one of the selected genes, min(*X*) is the minimum value, max(*X*) is the maximum value, and *Z* is the resulting variable that was used for the ML process.

Nowadays, there are a multitude of artificial intelligence techniques [23], and due to the difficulty of using all of them at the same time, we tried to use a representative selection of all of them in order to classify patients into the three categories previously described (DB, IR, or IS):

- The deep learning multilayer perceptron (MLP). It is a special type of network totally connected to multiple individual neurons. The input layer has the same number of inputs as the total of the predictor variables, in this case, 60. The middle layer looks for characteristics associated with the data. In this case, two intermediate layers were defined with 64 nodes. The output layer had the same number of outputs as the categories to predict, in this case, three. The activation function of the last layer was “softmax”, which converts a vector of values into a probability distribution. The loss function was “categorical crossentropy”, and accuracy was the metrics [24];
- K-nearest neighbor (kNN). This kNN algorithm begins with a training dataset made up of examples that are classified into several categories, as labeled by a nominal variable. Assume that there is a test dataset containing unlabeled examples that otherwise have the same features as the training data. For each record in the test dataset, kNN identifies k records in the training data that are the “nearest” in similarity, where k is an integer specified in advance. The unlabeled test instance is assigned the class of most of the k nearest neighbors [25]. Euclidean distance was used, and the k value was 7;
- Artificial neural network (ANN). The ANN uses a network of artificial neurons or nodes to solve learning problems. In this process, two neural network models were used. In the first one, the input data were the 60 genes obtained in the ADO process; two hidden layers were used, the first with 30 nodes and the second with 20 nodes. In the second, the input variables were the seven variables whose eigenvalues were higher or close to 1 in the principal components analysis carried out with the 60 genes. In this second model, two hidden layers were also used with five and three nodes, respectively. In both cases, the activation function was the logistic function (it is the main activation function and is very important since it can be derived), and the training algorithm was “backpropagation” [25];
- Support vector machine (SVM). A support vector machine (SVM) can be imagined as a surface that defines a boundary between various data points, representing examples plotted in multidimensional space according to their feature values. The goal of an SVM is to create a flat boundary, called a hyperplane, which leads to fairly homogeneous partitions of data on either side. When the data are not linearly separable, it is necessary to use kernels or similarity functions and specify a parameter C to minimize the cost function. The most popular kernels are the linear and the Gaussian [25]. In this analysis, the SVM technique was applied twice. The first has been linear (vanilladot option), and the second the Gaussian (rbfdot option). In both cases, the parameter C took the value 1;
- Random forest (RF). This technique combines versatility and power into a single machine learning approach. Because the ensemble uses only a small, random portion of the full feature set, random forests can handle extremely large datasets, where the so-called “curse of dimensionality” might cause other models to fail. At the same time, its error rates for most learning tasks are on par with nearly any other method. Individuals are selected at random with replacement, thus forming different data sets. Subsequently, a decision tree was created with each data set so that different trees were obtained. When creating the tree, the random variables in each node of the tree, and thus, without pruning the tree, were allowed to grow. Subsequently, the new data were predicted using the majority vote, classified as positive if the majority of trees predicted the observation as positive [25]. In this analysis, the random forest included 500 trees and tested seven variables in each division;
- Random forest by fivefold cross-validation (RF-5CV). The technique is RF, but in this case, the dataset has been split into five groups. Then, four folds were used as a training data set, and the remaining one was used for testing. This process was repeated for each of the five folders. This random forest model had 500 trees and tested two variables in each division.

With the intention of obtaining robust results, and not due to chance, the process of partitioning training data, test data, and the execution of the different techniques was repeated 1000 times, wherein a confusion matrix was calculated for each of them. The results of each execution were stored in a cumulative confusion matrix to evaluate it at the end of the 1000 executions. In the confusion matrix with three categories, the accuracy was calculated. In the confusion matrix where IR and DB were joined, the accuracy, sensibility (recall), specificity, positive predictive value (PPV or precision), and negative predictive value (NPV) were calculated. Increasing the sensibility may decline the PPV and vice versa. Increasing the specificity may decline the NPV and vice versa. The measurement metrics are explained below:

(2) $$Accuracy=\frac{TP+TN}{TP+TN+FP+FN}$$

(3) $$Sensibility/Recall=\frac{TP}{TP+FN}$$

(4) $$Specificity=\frac{TN}{TN+FP}$$

(5) $$Positive\;predictive\;value/Precision=\frac{TP}{TP+FP}$$

(6) $$Negative\;predictive\;value=\frac{TN}{TN+FN}$$

TP, TN, FP, and FN represent True positive, True negative, False positive, and False negative, respectively.

**Figure 2:**
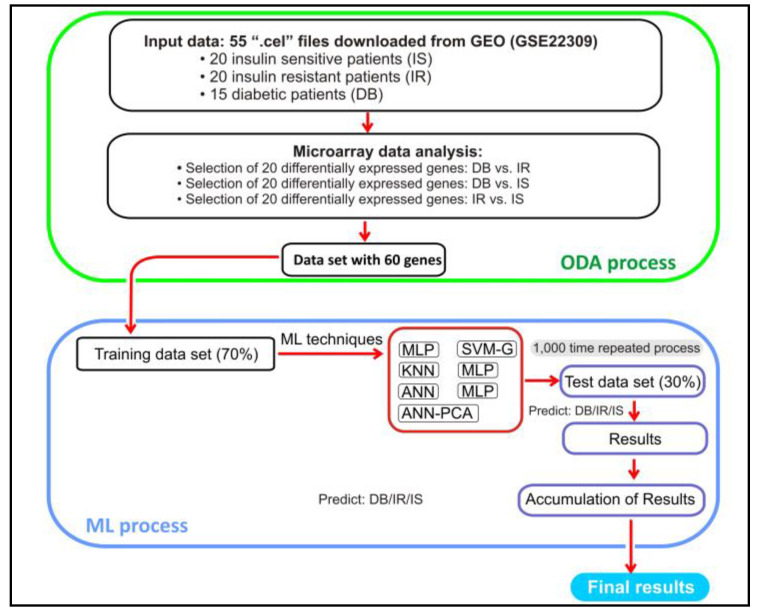
Workflow of the process.

The workflow of this study is exhibited in Figure 2.

## 3. Results

**Figure 3:**
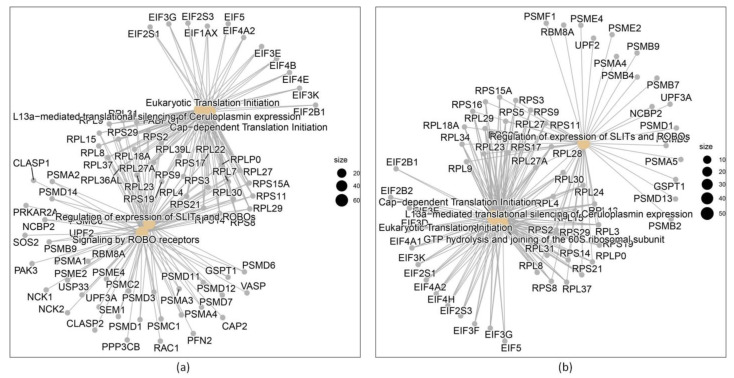
(**a**) Genes differentially expressed between DB vs. IS; (**b**) genes differentially expressed between IR and IS.

The results obtained in the process are shown sequentially. The original data set consists of 409,600 genes from 55 patients. Figure 3a,b suggest that there may be some factor that overlaps the differences between the groups. Since information on other covariates is not available, it is difficult to decide whether this is the case. A common cause of the batch effect is the date the samples are processed, but the hybridization date of all the records was not available either. After carrying out the data normalization process with the “rma” function, 12,626 genes from the 55 patients were selected.

After filtering the data, where 50% of the genes that present the greatest variability have been selected and that are also correctly annotated in the Entrez database, 4380 genes from the 55 patients were selected. After creating the design matrix and the contrast matrix, differentially expressed genes were selected in two-to-two comparisons.

Table 1 shows the 20 genes that present the smallest adjusted *p*-value ordered from lowest to highest for each comparison. These 60 genes (20 rows × 3 comparisons) have been the ones that will be used as the input file in the ML analysis.

Regarding multiple comparisons: (a) There were 294 genes differentially expressed between DB and IR, (b) there were 1843 genes differentially expressed between DB and IS, and (c) there were 863 genes differentially expressed between IR and IS.

Concerning the REACTOME process database, no relationships were found between the differentially expressed genes between the DB vs. IR groups; this may be because there were only 294 differentially expressed genes. In relation to the other two comparisons, only genes whose adjusted *p*-value was less than 0.05 were taken into account.

In relation to the other two comparisons, DB vs. IS and IR vs. IS, the five main processes related to differentially expressed genes are described in Table 2. More results related to the microarray process are shown in Appendix A.

In Figure 3, the networks produced by the differentially expressed genes are graphically detailed:

In relation to the prediction process with ML, once the file with the 60 genes described in Table 1 with 55 records was obtained, the target variable (DB, IR, and IS) was added. Then, the previously mentioned ML techniques are executed where the data set, after normalization, has been split randomly into training and test data. After executing the data set partition process and executing the techniques 1000 times, with the test data, a 3 × 3 confusion matrix was constructed for each technique where the accuracy was calculated.

Subsequently, in this confusion matrix, the IR and DB categories were unified (taking into account the similarity they present), and a new 2 × 2 confusion matrix was constructed where the accuracy, sensitivity, specificity, positive predictive value (PPV), and negative predictive value (NPV) were the measurement metrics taken into account to assess the performance of each technique. The results are shown in Table 3.

As the data are quite balanced (20 IS subjects, 20 IR subjects, and 15 DB subjects), accuracy is a good measurement metric in order to evaluate the results. The accuracy^a^ (3 groups) is greater than 80% in all cases, being greater than 90% with the MLP and linear-SVM techniques (95.42% and 90.99%, respectively). With these data, these two techniques achieve the best accuracy results. It may be surprising that RF and RF-5CV techniques are the ones that achieve the worst results in relation to accuracy. In any case, with these data, it is possible to make predictions with an accuracy of 95.42%. There are statistically significant differences between this result if we compare it with the accuracy of SVM-Lineal (95.42% vs. 90.99%, *p*-value < 0.001). If we look at the accuracy of unifying the IR and DB categories (two groups), it reaches 90% in all cases except RF, which is close to 90% (89.57%) and reaching 96.31% with MLP. As before, comparing with accuracy of SVM-Lineal, we can find statistically significant differences (96.31% vs. 94.53%, *p*-value < 0.001). The good behavior of all techniques can be observed, particularly the excellent results researched with MLP (deep learning).

Sensitivity was greater than 90% in all cases, being greater than 99.50% with the SVM-radial technique. In relation to specificity, the results vary more; the MLP technique is the one that presents a higher value with a value close to 94%, and the KNN technique is the one that presents the lowest value with a value around 77%. In relation to the PPV, the technique that presents the highest degree of success is once again MLP, with a value of around 96.6%, and the lowest is once again KNN, with a value of around 88%. Regarding the NPV, all the techniques present values close to 90% (ANN-PCA) or higher, with the SVM-radial technique reaching almost perfect prediction with a 99.00% prediction. In general, it can be stated that the accuracy achieved is very high in both cases.

Comparing our accuracy with other studies related to diabetes and machine learning, it can be seen that our predictions are superior to these studies. The Haewon Byeon article [26] presents an accuracy of 0.73. The Quincy A. Hathaway article [27] presents a testing accuracy of 0.778 using the Gaussian Naïve Bayes technique (Table 2 and Table 3). We have to be cautious with these comparisons since the methodologies used are not exactly the same.

## 4. Discussion

The spectrum of differentially regulated genes and the pathways in which the gene products are involved depends, among other factors, on the disease state, the evaluated disease phenotypes treatments, and/or the tissue or cell type evaluated [28]. Most of the studies focusing on DM found some targets related to the pathogenesis of the disease [28,29,30]. In accordance, we found that DEG genes implicated in pathways known to have a relevant role in DM onset and disease progression, as were numerous genes coding for transcription factors found to be dysregulated. However, Reactome pathway analysis for evaluated comparisons between DM and IS showed a significant contribution of genes in signaling mediated by the axon guidance proteins, Roundabout (Robo) receptors. Interestingly, proteome-wide Mendelian randomization and colocalization that evaluated the associations of blood proteins with DM risk and diabetic complications found ROBO2 as one of the proteins associated with the onset of DM. Another altered pathway represents the Cap-dependent translation that is initiated by the binding of the factor *eIF4E* to the cap domain of mRNA and the *L13a*–mediated translational silencing of ceruloplasmin expression.

Our study found differentially expressed genes among DB, IS, and IR patients, which showed high predictive value, obtaining an accuracy, sensitivity, and specificity of around 95% using the MLP technique.

This analysis is composed of two complementary working hypotheses, and we wanted to know if a certain number of differentially expressed genes in the comparison between the three groups (IS, IR, and DB) in a microarray analysis would subsequently be valid to predict the target variable composed of these three groups, using the said determined number of differentially expressed genes as predictive variables. Once the results have been analyzed, it can be stated that differentially expressed genes have been found between the three groups. It has also been possible to verify their biological significance, although in relation to the DB vs. IR comparison, no results have been obtained in the ReactomePA database, although results have been found in the GO database. Subsequently, after selecting the 20 differentially expressed genes for each one of the three comparisons with the lowest-adjusted *p*-value, predictive models were carried out in order to check whether these 20 genes are enough to predict with a high degree of validity of the target variable (IS, IR, and DB). In view of the results, it can be stated that these 60 genes serve to predict the target variable with great validity.

However, this study has two main limitations, and we must be cautious with the results obtained: (i) the typical problem of the Batch effect in the analysis of microarray data, (ii) the small sample size, only 55 individuals, used to perform the analysis of microarrays, and the subsequent use of machine learning techniques, which makes the study has little power.

On the other hand, once we have seen the graphs and results, it is worth commenting that the IR group is more similar to the DB group than the IS group.

## 5. Conclusions

In this analysis, the operation of the binomial between differentially expressed genes was proposed through an analysis of microarray data and the subsequent verification of the effectiveness of these data to make predictions using ML tools. Our results show that a certain number of genes are differentially expressed and serve to create predictive models with high validity. This would be interesting to be able to put into practice for the benefit of patients or people at risk of diabetes.

## Figures and Tables

**Table 1:** Differentially expressed genes with the smallest adjusted *p*-value used as predictor variables in the ML model.

N	DB vs. IR	DB vs. IS	IR vs. IS
1	*RAB11B*	*PCBD1*	*SAFB*
2	*TASOR*	*PCGF1*	*TNFAIP1*
3	*FAP*	*ATP1A3*	*NFIC*
4	*NEAT1*	*PRKAR2A*	*RAB31*
5	*LUM*	*ALOX12*	*CR1*
6	*VGLL1*	*ATP5ME*	*NFATC1*
7	*IKZF1*	*SLC22A6*	*RHOBTB2*
8	*ACSL4*	*GSPT1*	*PLD3*
9	*MPDZ*	*ACOX1*	*NUP188*
10	*XCL2*	*TFR2*	*RPS2*
11	*ACTL6A*	*SETBP1*	*RSU1*
12	*CACNA1G*	*EDA*	*BPTF*
13	*EPHX1*	*ATP5MC1*	*PIN1P1*
14	*KRT14*	*PRRC2C*	*MPP2*
15	*ARHGAP12*	*PPP2R5E*	*ZNF473*
16	*OGT*	*ATP5MC3*	*H4C3*
17	*NEDD4L*	*EXOC6B*	*APOA1*
18	*RAB11A*	*ZNF133*	*ATP6V1H*
19	*CDC27*	*MAP4*	*MAD2L1BP*
20	*PDE4A*	*SDCBP*	*TBC1D22A*

DB: diabetic patients; IR: insulin resistant patients; IS: insulin sensitivity patients.

**Table 2:** Relationships between the differentially expressed genes.

DB vs. IS	IR vs. IS
Signaling by ROBO receivers	Eukaryotic Translation Initiation
Regulation of expression of SLITs and ROBOs	Cap-dependent Translation Initiation
Eukaryotic Translation Initiation	GTP hydrolysis and joining of the 60S ribosomal subunit
Cap-dependent Translation Initiation	L13a-mediated translational silencing of Ceruloplasmin expression
L13a-mediated translational silencing of Ceruloplasmin expression	Regulation of expression of SLITs and ROBOs

DB: diabetic patients; IR: insulin-resistant patients; IS: insulin-sensitivity patients.

**Table 3:** Results of applying ML techniques.

Technique	Accuracy^a^ *	Accuracy^b^ *	Sens.	Spec.	PPV	NPV
MLP	95.42	96.31	97.65	93.94	96.58	95.8
KNN	85.51	90.65	98.49	76.88	88.2	96.68
ANN	88.34	92.96	96.04	87.57	93.13	92.65
ANN-PCA	89.01	91.99	93.32	89.65	94.05	88.44
SVM-radial	89.55	93.06	99.54	81.69	90.51	99.02
SVM-lineal	90.99	94.53	97.09	90.04	94.47	94.64
RF	80.97	89.57	95.14	79.8	89.2	90.35
RF—5CV	81.92	90.29	96.11	80.05	89.44	92.14

* Accuracy^a^ is the accuracy of the 3 × 3 confusion matrix, and accuracy^b^ is the 2 × 2 confusion matrix after unified DB and IR. Sens. is sensitivity, and Spec. represents specificity. MLP: multilayer perceptron technique; KNN: K-nearest neighbor; ANN: artificial neural network; ANN-PCA: artificial neural network using seven variables obtained from the PCA process; SVM-radial: support vector machine the Gaussian kernel; SVM-lineal: support vector machine using the linear kernel; RF: random forest technique; RF-5CV: random forest technique using 5-fold cross-validation.

## Data Availability

The data have been obtained from the platform Gene Expression Omnibus (GEO) with the identifier “GSE22309” and whose title is “Expression data from human skeletal muscle”. Stimulate files have been used.

## Appendix A

This section shows results from the microarray process that has not been included in the Section 3. Firstly, the box plots of the intensity of the values, the signal density plot of the data distribution, and the two-dimensional plot of the principal component analysis of the raw data are shown in Figure A1.

In Figure A2, the three graphs are shown again after normalizing the data. It can be said that normalization has worked correctly.

In Figure A3, the distribution of gene variability is analyzed. The plot shows that the most variable genes are those with a standard deviation above 90–95% of all standard deviations. This figure presents the usual appearance.

Below are the “volcano plots” for each of the comparisons. The names of the five genes with the smallest-adjusted *p*-value are shown. You can see which genes are over-expressed or under-expressed.

Through a “heatmap”, the expressions of each gene can be visualized, grouping them to highlight the genes that are “up” or “down” regulated simultaneously, constituting expression profiles. Figure A5 shows the “heatmap” with the 60 genes used in the ML process. It can be seen that the model discriminates clearly between the IS (green), IR (blue), and DB (red) categories.

In relation to the GO database, searches have been carried out for both over-expressed and under-expressed genes, resulting in six files in HTML format. The first three links of each result are linked in Table A1.

**Table A1:** Processes obtained from the GO database based on differentially expressed genes.

Comparison	Process
DB vs. IR Over-representation	spleen development
	embryonic heart tube left/right pattern formation
	left/right pattern formation
DB vs. IR Under-representation	mRNA catabolic process
	RNA catabolic process
	translation
DB vs. IS Over-representation	mRNA metabolic process
	regulation of translation
	posttranscriptional regulation of gene expression
DB vs. IS Under-representation	extracellular matrix organization
	extracellular structure organization
	external encapsulating structure organization
IR vs. IS Over-representation	translational initiation
	SRP-dependent cotranslational protein-targeting membrane
	nuclear-transcribed mRNA catabolic process, nonsense-mediated decay
IR vs. IS Under-representation	external encapsulating structure organization
	urogenital system development
	extracellular matrix organization
